# Removal of organic contaminants in bioretention medium amended with activated carbon from sewage sludge

**DOI:** 10.1007/s11356-017-9508-1

**Published:** 2017-06-29

**Authors:** Karin Björklund, Loretta Li

**Affiliations:** 1 Department of Civil and Environmental Engineering, Chalmers University of Technology, Sven Hultins Gata 8, SE-41296 Goteborg, Sweden; 2 Department of Civil Engineering, University of British Columbia, 6250 Applied Science Lane, Vancouver, BC V6T 1Z4 Canada

**Keywords:** Bioretention, Column test, Dissolved organic matter, Hydrophobic organic pollutants, Rain garden, Sorption, Soil amendment, Stormwater management

## Abstract

Bioretention, also known as rain garden, allows stormwater to soak into the ground through a soil-based medium, leading to removal of particulate and dissolved pollutants and reduced peak flows. Although soil organic matter (SOM) is efficient at sorbing many pollutants, amending the bioretention medium with highly effective adsorbents has been proposed to optimize pollutant removal and extend bioretention lifetime. The aim of this research was to investigate whether soil amended with activated carbon produced from sewage sludge increases the efficiency to remove hydrophobic organic compounds frequently detected in stormwater, compared to non-amended soil. Three lab-scale columns (520 cm^3^) were packed with soil (bulk density 1.22 g/cm^3^); activated carbon (0.5% *w*/*w*) was added to two of the columns. During 28 days, synthetic stormwater—ultrapure water spiked with seven hydrophobic organic pollutants and dissolved organic matter in the form of humic acids—was passed through the column beds using upward flow (45 mm/h). Pollutant concentrations in effluent water (collected every 12 h) and polluted soils, as well as desorbed amounts of pollutants from soils were determined using GC-MS. Compared to SOM, the activated carbon exhibited a significantly higher adsorption capacity for tested pollutants. The amended soil was most efficient for removing moderately hydrophobic compounds (log *K*_ow_ 4.0–4.4): as little as 0.5% (*w*/*w*), carbon addition may extend bioretention medium lifetime by approximately 10–20 years before saturation of these pollutants occurs. The column tests also indicated that released SOM sorb onto activated carbon, which may lead to early saturation of sorption sites on the carbon surface. The desorption test revealed that the pollutants are generally strongly sorbed to the soil particles, indicating low bioavailability and limited biodegradation.

**Electronic supplementary material:**

The online version of this article (doi:10.1007/s11356-017-9508-1) contains supplementary material, which is available to authorized users.

## Introduction

Surface runoff from urban areas is a primary cause of flooding and erosion of urban streams, as well as a major source of pollution of receiving water bodies. Bioretention and rain gardens are examples of green infrastructure that help maintain or restore the natural flow pattern of stormwater by collecting and storing the water, allowing it to infiltrate into the ground, removing pollutants and recharging the groundwater table and natural streams (European Commission 2013; US EPA 2014; Waterbucket 2014). Bioretention and rain garden are used interchangeably, but bioretention tends to be used for larger-scaled systems with more detailed design criteria. Both systems consist of a shallow, planted depression in the landscape that allows surface runoff from impervious areas to soak into the ground through a medium, usually a native or engineered soil. This reduces peak flows and flow volume, as well as promoting removal of both particulate and dissolved contaminants in stormwater (Davis et al. 2009; Grebel et al. 2013; LeFevre et al. 2014; Muthanna et al. 2007; Trowsdale and Simcock 2011). Metals such as Cu and Zn occur in both particulate and dissolved form in polluted stormwater (Camponelli et al. 2010; Morrison et al. 1990). Recent studies show that hydrophobic organic pollutants, including, e.g. polycyclic aromatic hydrocarbons (PAHs), are also present in the colloidal and dissolved phase in stormwater (Brown 2002; Kalmykova et al. 2013; Kalmykova et al. 2014; Zgheib et al. 2011). Studies indicate that, aside from particle sedimentation, sorption followed by biodegradation are the major hydrocarbon removal mechanisms in bioretention and rain gardens, although volatilization and plant uptake are also possible pathways (LeFevre et al. 2014; LeFevre et al. 2012a; Zhang et al. 2014). Given that sorption is a major removal process for organic pollutants, the performance of the bioretention medium is essential. Currently, there are very few performance data for bioretention and rain gardens, especially related to the removal of organic compounds (Davis et al. 2009; DiBlasi et al. 2008; Grebel et al. 2013; LeFevre et al. 2012a; Zhang et al. 2014). Existing studies indicate that these systems are effective in removing organic pollutants from stormwater; for hydrophobic compounds such as petroleum hydrocarbons and lighter PAHs, 80–90% reduction of the incoming load can be expected (DiBlasi et al. 2008; Hong et al. 2006; LeFevre et al. 2012a).

To further enhance the removal of pollutants from stormwater, amending the bioretention media with biochar, fly-ash and water treatment residuals has been proposed (O’Neill and Davis 2012; Tian et al. 2014; Zhang et al. 2008). However, studies on media amendment to enhance the removal of organic pollutants are to date very limited. The hypothesis is that media amended with efficient adsorbents exhibit limited pollutant migration, and delayed saturation and loss of sorption capacity compared to non-amended media. This would lead to lower media replacement frequency, hence lower costs of maintenance. Contaminated soils amended with activated carbon and biochar exhibit reduced mobility and bioaccumulation of metals (e.g. As, Cd, Pb and Zn) and organic compounds (e.g. PAHs and the herbicide atrazine) compared to non-amended soils (Beesley and Marmiroli 2011; Bian et al. 2014; Jakob et al. 2012; Wang et al. 2014; Ulrich et al. 2015). In previous research (Björklund and Li 2017), we have shown that activated carbon produced from domestic sewage sludge is an efficient adsorbent for removing hydrophobic organic compounds (HOCs) such as PAHs, phthalates and alkylphenols.

Although activated carbon is very efficient for removal of HOCs from water, the presence of dissolved organic matter (DOM) in stormwater may negatively affect HOC sorption through competition for sorption sites on the adsorbent surface and blockage of pores by the large DOM molecules (Kilduff et al. 1996; Li et al. 2003; Pelekani and Snoeyink 1999; Quinlivan et al. 2005). In previous tests of sewage sludge-based activated carbon (SBAC), batch sorption tests were deemed ineffective due to low water solubility of HOCs and high sorption capacity of SBAC, leading to low saturation of the carbon and little competition between HOCs and DOM. In continuous flow systems, such as column studies, the sorbent is initially only partially loaded with HOCs and DOM, but more HOCs and DOM continuously enter the system, possibly leading to competition for adsorption sites (Li et al. 2003). Testing SBAC in column studies where a continuous inflow of both HOCs and DOM may affect the sorbents’ performance is therefore necessary.

The aim of this research was to investigate whether a soil-based bioretention medium amended with SBAC increases the removal efficiency of frequently detected HOCs in stormwater, compared to non-amended medium. The scope of this study is limited to sorption of dissolved HOCs; removal of particle-bound pollutants is excluded. The objectives of the study were to (i) compare the adsorption capacity of SBAC and soil organic matter (SOM) from bioretention medium in batch sorption tests; (ii) compare the removal of HOCs in bioretention medium with and without addition of SBAC using column tests; and (iii) determine the desorption of HOCs from polluted bioretention medium. These objectives are based on the following hypotheses: (i) organic matter in soil, usually 0–5% *w*/*w* in bioretention media (Fassman et al. 2013; Rouhi and Schwartz 2007), is often efficient at capturing HOCs and other pollutants (Chefetz et al. 2000; Chiou et al. 1983). However, adsorption onto SBAC is hypothesized to be higher than to SOM due to the large surface area of SBAC (approximately 700 m^2^/g [Björklund and Li 2017]) and microporosity, which is suitable for HOC adsorption (Smith et al. 2009). (ii) Due to the high sorption capacity of SBAC (Björklund and Li 2017), non-amended medium will reach breakthrough of HOCs prior to SBAC-amended medium; and (iii) sorption of HOCs to soil is reversible, and sorbed pollutants may be desorbed due, for example, to changes in soil properties and co-transport with colloidal matter (Badea et al. 2013; Durjava et al. 2007).

## Materials and methods

### Studied compounds

Screening studies indicate that PAHs, phthalates and alkylphenols are ubiquitous in urban runoff and frequently detected in concentrations that exceed European as well as Canadian water quality standards (Björklund et al. 2009; Björklund et al. 2011; Bressy et al. 2012; Clara et al. 2010; Zgheib et al. 2011). Specific organic compounds included in this study are as follows: three PAHs (fluorene, anthracene and pyrene; 2.5, 3 and 4 aromatic rings, respectively); two alkylphenols (4-nonlyphenol and 4-*tert*-octylphenol); and two phthalates (dibutyl phthalate and di(2-ethylhexyl) phthalate). Selected physical-chemical properties of the compounds are presented in Table 1.

**Table 1:** Acronyms, CAS identification numbers and selected physico-chemical properties of the organic compounds studied

Chemical name (acronym)	CAS number	MW (g/mol)	Water solubility^a^ (mg/l)	Log *K*_ow_^b^	*K*_*oc*_^b^
Polycyclic aromatic hydrocarbons					
Fluorene (FL)	86-73-7	166	1.8	4.02	4241
Anthracene (ANT)	120-12-7	178	1.3	4.35	7274
Pyrene (PYR)	129-00-0	202	7.7 × 10^−2^	4.93	17,180
Phenols					
4-*tert*-Octylphenol (OP)	140-66-9	206	19	4.12	10,290
4-Nonylphenol (NP)	84852-15-3	220	5.4	4.48	18,970
Phthalates					
Dibutyl phthalate (DBP)	84-74-2	278	9.9	4.57	1919
Di(2-ethylhexyl) phthalate (DEHP)	117-81-7	391	2.5 × 10^−3^	7.50	99,470

^a^DEFRA 2008; ECB 2002, 2004, 2008; Mackay et al. 2006

^b^Log *K*_ow_ values from KOWWIN™ (v1.68) and *K*_*oc*_ from KOCWIN™ of the EPI Suite™ (US EPA 2012)

### Bioretention medium

The medium was sampled from a rain garden receiving runoff from a roof and minor surface runoff from a grassed area. The rain garden had been in operation for 1.5 years at the time of sampling. Samples were taken from approximately 50–300 mm below the surface using a clean pail and shovel. The medium, henceforth referred to as soil, an engineered soil marketed under the name Cascade Ecomedia, is developed by Cascade Envirotech (Aldergrove, BC, Canada). This soil was stored in darkness at 4 °C prior to use in our experiments. In the sieved soil (<2 mm), almost 90% of the soil particles are ≤250 μm, pH = 4.3, particle density = 2.54 g/cm^3^ and loss on ignition = 3.75% (more details in Supplementary Material and Björklund and Li 2016). Leaching tests (Björklund and Li 2016) revealed that none of the studied HOCs was released from the soil in a detectable amount.

### SBAC

The production of SBAC and the material’s characteristics are described in detail in Gong (2013) and Björklund and Li (2017). The carbon source is dried sludge from a domestic sewage treatment pilot plant. The sludge was soaked in the activation agent ZnCl_2_, and carbonized through pyrolysis at *T*_final_ = 500 °C. The finished carbon product was ground and washed with HCl and distilled water before final drying (105 °C) and storage in amber glass bottles. The pH, surface area and ash content of the activated carbon were 3.4, 700 m^2^/g and 9%, respectively (Björklund and Li 2017).

### Chemical standards

Standards of 4-nonylphenol (85% content of *para*-isomers), fluorene, anthracene, pyrene, dibutyl phthalate, di(2-ethylhexyl) phthalate, acenaphthene-d_10_, phenol-d_6_, benzyl benzoate and phenanthrene-d_10_ and humic acids (HAs) were purchased from Sigma-Aldrich, and 4-*tert*-octylphenol was supplied by Fluka. Stock standard solutions of the organic compounds were prepared in toluene obtained from Caledon Laboratories Ltd. (final concentration approximately 5000 ng/μL), and working standards for spiking water samples were prepared in acetone from Fisher Scientific (final concentration approximately 100 ng/μL). Acenaphthene-d_10_ and benzyl benzoate were used as surrogate standards, whereas phenol-d_6_ and phenanthrene-d_10_ served as internal standards. Internal and calibration standards of analytes were prepared in toluene. All solutions were stored in amber glass bottles at −18 °C until use.

### Extraction and analysis of organic compounds in water samples

The liquid–liquid extraction (LLE) of HOCs in water is described in detail in Björklund and Li (2015). In short, the HOCs were simultaneously extracted with 3 × 25 mL dichloromethane (DCM, from Fisher Scientific), evaporated to dryness with N_2_, and the extracts were reconstituted with toluene. Extraction of HOCs from soils is described in the “Determination of HOC loads in polluted soils” section.

Internal standards (phenol-d_6_ and phenanthrene-d_10_) were added to extracts and calibration solutions before GC-MS analysis. The analytical procedure using GC-MS was similar for both water and soil samples and is described in detail in Björklund and Li (2015). Compounds were identified using gas chromatographic retention times and mass spectral patterns of at least three ions. For quantification, six-point calibration curves were prepared for all target compounds. The concentration range for samples from the batch sorption tests (see the “Batch sorption tests of SBAC and SOM” section) was 2.5–150 and 2.5–50 μg/L for water and soil samples from the column tests, respectively (see the “Column tests” and “Determination of HOC loads in polluted soils” sections, respectively).

### Batch sorption tests of SBAC and SOM

To compare the sorption capacity of SBAC and SOM, batch sorption tests were performed. The tests were performed similarly to a previous study on SBAC adsorption of HOCs (Björklund and Li 2017) to achieve comparable results. One hundred and fifty millilitres of ultrapure water (purified with a Synergy UV Milli-Q system from Millipore) was spiked with a mixture of the seven HOCs (Table 1) to final concentration 100 μg/L of each compound. Fifteen milligrammes of SBAC or SOM was contacted with the HOC solution for 24 h in an end-over-end tumbler. In the SOM samples, a soil mass corresponding to 15 mg OM (derived from the determined organic content, see Supplementary Material) was used. The solid phase was removed by centrifugation at 2000×*g* for 10 min. The liquid phase was extracted and analysed by GC-MS (see the “Extraction and analysis of organic compounds in water samples” section). All samples were made in duplicates. For all batches, matrix blanks and a matrix spike were prepared following the same procedure to determine contamination and loss of analytes, respectively.

### Column tests

Three columns were used in the continuous flow tests: two columns with soil amended with 0.5% (*w*/*w*) SBAC and one control column with non-amended soil. The chosen amendment dose has been used by other researchers, e.g. Tian et al. (2014). To ensure a homogenous material, the soil was sieved to remove particles >2 mm in nominal size and long plant fibres. Sorption onto the medium occurs during infiltration of stormwater and is a kinetic process, therefore dependent on medium–pollutant contact time, i.e. infiltration rate. Typical bioretention and rain garden infiltration rates are 50–200 mm/h (generally approaching 100 mm/h) and soil dry bulk densities are 0.8–2.0 g/cm^3^ (generally 1.5 g/cm^3^) (Greater Vancouver Sewerage and Drainage District 2012; KWL 2012; Rouhi and Schwartz 2007). The soil bulk density was determined according to the cylindrical core method (D7263 [ASTM 2009]) and the moisture content according to D2216 (ASTM 2010). Particle density (specific gravity) was determined using the pycnometer method and volumetric flask method (D854 [ASTM 2014]). All parameters were determined on triplicate samples.

All column components were stainless steel. The interior measures of the columns were as follows: diameter 114 mm and height 51 mm. A detailed description and schematic of the columns are found in Li (2011). Prior to use, all components of the column set-ups were washed with detergent and water, followed by acetone rinsing. Height-to-width ratio <1 was chosen because increasing the width of the column can reduce the potential of preferential and sidewall flows, which easily occur in columns with high height-to-width ratios, leading to poorer contact between solid material and synthetic stormwater. To further prevent sidewall leakage, the inner walls of the columns were smeared with a thin layer of bentonite slurry (moisture content ~200%). Porous stones were used at the top and bottom of the columns to prevent preferential flow. The stones were cleaned by sonicating in ultrapure water for 1 h at room temperature and then boiled for 30 min to remove air pockets. The soil was compacted in the columns according to D698 (ASTM 2012). The resulting bulk density in the columns was 1.22 g/cm^3^, which is within the range, but on the lower end, of observed densities in bioretention and rain gardens. From the bulk and particle density (2.54 g/cm^3^), the average pore volume was estimated to be 227 cm^3^ (Budhu 2007). The SBAC was evenly spread on top of the packed soil layer (i.e. closest to the outlet) in two columns.

The columns were operated at constant upward water flow, which generally leads to a more even flow than downward flow column leaching. The system was fed with synthetic stormwater: to tap water, a humic acid stock was added to a final dissolved organic carbon (DOC) concentration of approximately 20 mg/L, and the working standard of HOCs was added to a final concentration of 50 μg/L of each of the seven HOCs. The synthetic stormwater was prepared twice a day in three pressurized stainless steel containers in series, with a total volume of 20 L (gravity flow could not be utilized because of practical reasons). The flow rate of the system was controlled by three peristaltic pumps in parallel, connected to the stainless steel containers and the inlet of the columns using Teflon tubing. Each pump was set to 6.4 mL/min, resulting in an infiltration rate of 44.7 mm/h. The rate is slightly lower than design recommendations (50 mm/h), but was determined by the total volume of the stainless steel containers. Teflon tubing was also used at the outlet of the columns to drain effluent water. Effluent water samples were collected in amber glass bottles every 12 h (corresponding to every 20 pore volume) during 35 min, each sample corresponding to one pore volume. Samples were stored in a cold room at 4 °C for no longer than 4 days until extraction and GC-MS analysis. For all batches, a matrix spike and a blank were prepared and analysed together with column eluate samples. The experiment was terminated after 28 days, corresponding to 1000 pore volumes, of continuous flow.

Eluate samples from the column study were analysed for HOCs using GC-MS (see the “Extraction and analysis of organic compounds in water samples” section) and DOC using a Lachat Instrument IL 500 TOC analyser. This instrument was later replaced by a Dohrmann Phoenix 8000, UV-Persulfate TOC Analyser (see the “The effect of dissolved organic matter on sorption capacity” section).

### Desorption of HOCs from polluted soils

After completion of the column study, soil from the control column was divided into three equally thick (17 mm) layers. These individual portions were thoroughly mixed prior to moisture content analyses (triplicates of each layer). Desorption of leachable HOCs from each layer was determined following the ISO/TS 21268-2 standard method (International Organization for Standardization 2007). After 24 h agitation, the samples (triplicates of each layer) were settled for 10 min, centrifuged at 2000×*g* for 10 min and filtered through a 934-AH glass fibre filter (Whatman). The HOCs were extracted from soil eluates using the LLE method described in the “Extraction and analysis of organic compounds in water samples” section.

### Determination of HOC loads in polluted soils

The sorbed loads of HOCs in soil were determined on triplicate samples of each layer of the control column soil (see the “Desorption of HOCs from polluted soils” section). The concentration of HOCs (μg/g) in each layer was multiplied by the known mass of dry soil (g) in each layer to achieve the total HOC load (mg) in the control column soil. Extraction of HOCs from soil was based on methods presented by Hollender et al. (2003) and Song et al. (2002). Approximately 2 g of the moist soil was spiked with 1.0 mL surrogate standard (acenaphthene-d_10_ and benzyl benzoate in acetone), after which 5 mL acetone and 25 mL DCM were added to the vial. The samples were manually shaken for 1 min and vented to release potential excess gas. The samples were then placed in an ultrasonic bath (40 °C, VWR Scientific Products, Aquasonic, model 50 HT, 35 kHz) for 2 h and inverted every 10 min. The extracts were filtered through 1.5 g anhydrous Na_2_SO_4_ (placed in baked glass fibre filter from VWR, 1.6 μm pore size), evaporated to a few millilitres using a rotavaporator and then, to complete dryness, with a gentle stream of N_2_. The extracts were reconstituted with 1.00 mL toluene and internal standard (phenanthrene-d_10_ and phenol-d_6_) before analysis by GC-MS (same procedure as for water samples). The background concentrations of HOCs in unpolluted soil were determined on duplicate non-spiked soil samples. A solvent blank (no soil) was also extracted using the same procedure.

The efficiency of the extraction was tested by spiking triplicate blank samples (unpolluted soil) with all HOCs in acetone (final concentration 20 μg/g of each HOC). The spiked samples were dried at room temperature before addition of surrogates and initiation of the extraction procedure. The average recoveries of acenaphthene-d_10_ and benzyl benzoate were 64 and 112%, respectively. In the spiked samples the recoveries of HOCs were as follows: fluorene 47, anthracene 75, pyrene 84, octylphenol 86, nonylphenol 114, dibutyl phthalate (DBP) 102 and di(2-ethylhexyl) phthalate (DEHP) 141%. The low recoveries of acenaphthene-d_10_ and fluorene may be due to their higher volatility, whereas the high recovery of DEHP may have been caused by contamination problems.

### Data analysis

#### Statistical analyses

The program IBM SPSS Statistics 22 was employed to perform statistical analyses, including Pearson’s and Spearman’s correlations and Mann–Whitney *U* test. Pearson’s correlation represents a linear relationship between two variables and requires normally distributed data. Spearman’s rank correlation works by converting each variable into ranks, and is used when data do not meet the assumptions about normality and linearity. Because the logarithm of the partitioning coefficient *K*_ow_ is used, correlations involving *K*_ow_ are tested using the rank test. The Mann–Whitney *U* test is used to compare differences between two independent groups when data are not normally distributed. Normality was tested using the Shapiro–Wilk test: if *p* > 0.05, the distribution of data is not significantly different from a normal distribution.

#### Sorption capacity

In the batch tests, the sorption capacity at equilibrium, *q*_*e*_ (μg/g), was determined using

1 $$ {q}_e= V\times \frac{C_0-{C}_e}{m} $$

where *C*_0_ and *C*_e_ are the initial and equilibrium concentrations of the HOCs in the water phase (μg/L), respectively; *V* is the volume of the solution (L); and *m* is the weight of the adsorbent (g).

#### Breakthrough curves

The outcomes of column studies are often reported using breakthrough curves: a plot with time (*t*) or pore volume (*V*) on the *x*-axis and *C/C*_0_ on the *y*-axis, where *C* is the HOC concentration in a sample at a specific time or pore volume and *C*_0_ is the initial HOC concentration (50 μg/L in this study). The breakthrough time is arbitrarily set; in the current study, we use *C*/*C*_0_ = 0.10, i.e. 10% of the initial concentration is detected in the effluent.

#### Estimation of enhanced pollutant removal using SBAC

One of the hypotheses behind this study is that amendments extend the lifetime of the medium before saturation occurs and pollutant leaching becomes unacceptable. In this example, we used fluorene to calculate the enhanced sorption capacity brought by SBAC, as compared to non-amended soil.

From the column effluent concentrations and applied volume of synthetic stormwater, the difference in removed fluorene loads between the control and SBAC-amended columns was calculated. The SBAC and soil mass in the column were then scaled up to match the SBAC and medium mass in 1 m^2^ bioretention or rain garden. The assumed medium depth is 0.5 m (Fassman et al. 2013; KWL 2012), i.e. 1 m^2^ bioretention or rain garden corresponds to 0.5 m^3^ soil. The fluorene load removed by 0.5% (*w*/*w*) SBAC amended to 1 m^2^ bioretention (0.5 m^3^) was then calculated.

In residential areas, each square metre of bioretention or rain garden is usually connected to a larger contributing watershed than roads with high traffic count, due to the lower percentage of impervious surface. On the other hand, pollutant concentrations are generally lower in residential than in heavily trafficked areas. Two scenarios were therefore simulated: (1) The bioretention receives runoff from a residential area, where the concentration of fluorene is low (0.05 μg/L), the runoff coefficient ɸ = 0.35 and the size of the bioretention surface area is 2% of the impervious area that drains into the bioretention; and (2) the bioretention receives runoff from a traffic area, where the concentration of fluorene is high (1 μg/L), ɸ = 0.8 and the size of the bioretention surface area is 10% of the impervious area that drains into the bioretention. Data on fluorene concentrations, runoff coefficients and impervious/pervious area were obtained from the StormTac database (2016), Tegelberg and Svensson (2013) and Campbell et al. (2013), respectively. Using these assumptions, together with the annual rainfall in Langley, BC, Canada (1375 mm), the annual runoff volumes and corresponding fluorene loads drained to 1 m^2^ bioretention in a residential and a traffic area were estimated. Ultimately, the annual fluorene loads estimated from the two scenarios were compared to the removed mass of fluorene in 1 m^2^ bioretention amended with 0.5% SBAC.

#### Theoretical sorption, soil and water distribution estimations

The theoretical maximum mass, *m*_*ads*_ (mg), of HOCs that can be sorbed to the soil is calculated using:

2 $$ {m}_{ads}={K}_{oc}\times {f}_{oc}\times {m}_{soil} $$

where *K*_*oc*_ is the organic carbon-normalized partition coefficient between soil and water phases; *f*_*oc*_ is the mass fraction of soil organic matter content (0.0375); and *m*_*soil*_ is the soil mass (g) in the column. *K*_*oc*_ is a literature value derived from *K*_ow_ (Table 1).

The desorption coefficient, *K*_*des*_ (L/kg), is the ratio between the content of the HOC remaining in the soil phase and the mass concentration of the desorbed compound in the aqueous solution, calculated from

3 $$ {K}_{des}=\frac{m_s^{ads}-{m}_{aq}^{des}}{m_{aq}^{des}}\times \frac{V_T}{m_{s oil}} $$

where ${m}_s^{ads}$ (μg) is the mass of the test compound adsorbed on soil at adsorption equilibrium (i.e. adsorbed load at termination of column experiment); ${m}_{aq}^{des}$ (μg) is the mass of the test compound desorbed from soil at desorption equilibrium (i.e. desorbed load at termination of desorption test); *V*_*T*_ (L) is the volume of aqueous phase in contact with the soil during the desorption test; and *m*_*soil*_ (g) is the mass of soil used in the desorption test (OECD 2000). A high *K*_*des*_ value means that the substance is strongly sorbed to soil and organic matter, whereas a low value means that the compound is highly mobile in soil.

## Results and discussion

### Batch sorption tests of SBAC and SOM

The sorption of HOCs contacted with SOM was substantially lower than with SBAC. The differences between initial (*C*_0_) and final concentrations (*C*_*e*_) of the phthalates DBP and DEHP in batch tests with soil were only a few microgrammes per litre, i.e. very low sorption capacity. The soil’s sorption capacities for nonylphenol and octylphenol were almost five (*q*_*e,SOM*_ = 200 μg/g) and eight (*q*_*e,SOM*_ = 120 μg/g) times lower, respectively, than SBAC’s capacity (*q*_*e,SBAC*_ ≥900 μg/g for both alkylphenols) (Björklund and Li 2016). The average adsorption capacities for all HOCs onto SOM were significantly lower than onto SBAC (Mann–Whitney *U* = 1.00, *p* = 0.003, two-tailed). The highest removal using soil was observed for the PAHs, with 70% of pyrene being removed, corresponding to *q*_*e,SOM*_ = 660 μg/g, whereas approximately 30% of fluorene and anthracene were removed, corresponding to *q*_*e,SOM*_ = 270 and 200 μg/g, respectively. These results suggest that SOM has potential to sorb large quantities of HOCs, due to its sorption capacity and abundance (≤5% *w*/*w*) in some engineered soils used as bioretention medium. In fact, SOM is dominated by apolar groups, such as aliphatic and aromatic moieties, which are important sorption sites for HOCs (Kyoichi 1987). However, the sorption capacity of SBAC is superior to that of SOM, due to the abundance of hydrophobic sites and high surface area (Björklund and Li 2017). Hence, SBAC has the potential to add supplementary pollutant removal and storage if amended to bioretention and rain garden soils.

### Removal of HOCs in column tests

#### Breakthrough curves

**Fig. 1:**
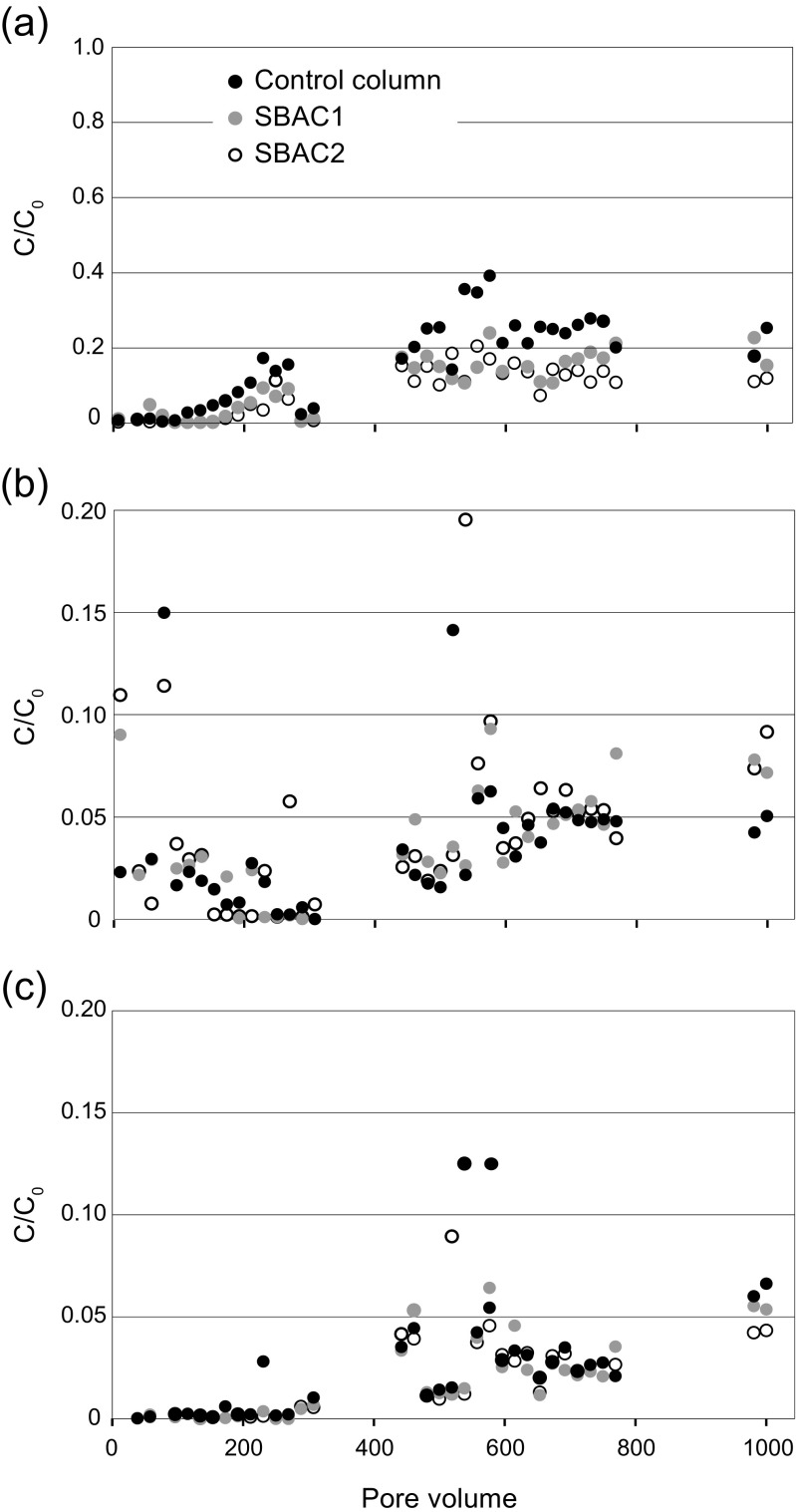
Breakthrough curves of **a** fluorene (log *K*_ow_ = 4.02), **b** nonylphenol (log *K*_ow_ = 4.48) and **c** pyrene (log *K*_ow_ = 4.93) indicating the effluent–inlet concentration ratio (*C*/*C*_0_) as a function of the number of pore volumes passed through two columns with added SBAC (0.5% *w*/*w*) and the control column. Each pore volume corresponds to 227 mL. Note the different scales on the *y*-axis

Breakthrough (*C*/*C*_0_ = 0.10) was reached at very different pore volumes for different compounds. As a rule, the least hydrophobic compound of each group—fluorene, octylphenol and DBP (log *K*_ow_ = 4.02–4.57)—exhibited shorter breakthrough times and were observed in higher concentrations in the effluent than the more hydrophobic compounds. This trend is illustrated in the breakthrough curves of fluorene, nonylphenol and pyrene (Fig. 1). Breakthrough curves of anthracene, octylphenol and DBP are found in the Supplementary Material, Fig. S2. Breakthrough of fluorene was reached at 200 pore volumes (Fig. 1a), after which effluent concentrations ranged between 10 and 30% of the influent concentration (50 μg/L). Octylphenol reached *C*/*C*_0_ = 0.10 within 100 pore volumes in all three columns, which is the shortest breakthrough time of all compounds tested (Fig. S2). The effluent concentrations of DBP exhibited great variations; breakthrough was reached after approximately 180 pore volumes, and reached *C*/*C*_0_ = 0.30–0.40 between pore volumes 450 and 650, but was subsequently stabilized at *C*/*C*_0_ = 0.05–0.20, with the higher effluent concentrations found for the control column (Fig. S2). Octylphenol showed similar behaviour to DBP from pore volume 450 and onwards. The time-limited increase in effluent concentrations of DBP and octylphenol may depend on required equilibration time before sorption reached its capacity, as previously reported, e.g. by Ulrich et al. (2015). During the entire course of the experiment, breakthrough (0.10) was never reached (outliers excepted) for anthracene (Fig. S2), pyrene and nonylphenol (Fig. 1a, c). The phthalate DEHP, which is the most hydrophobic compound studied (log *K*_ow_ = 7.5), exhibited inconclusive results. This could be a result of contamination of samples, which is commonly observed for phthalates due to their ubiquitous occurrence in plastic materials. In addition, because of the low water solubility of DEHP (Table 1), this substance may attach to labware surfaces or form emulsions in water, which is not evenly distributed in the columns, leading to variations in influent and effluent concentrations (Julinová and Slavík 2012). Accordingly, DEHP was excluded from further water data analysis. In general, the average *C*/*C*_0_ (all pore volumes) for HOCs in all three columns correlated well with compound log *K*_ow_ (Spearman’s *ρ* = −0.638 to −0.725).

The difference in effluent concentrations was not statistically different between the two columns with added SBAC (Mann–Whitney *U* = 545–626, *p* = 0.140–0.804, two-tailed). However, effluent concentrations of fluorene, octylphenol and anthracene were significantly higher in the control column than in the two columns with SBAC (Mann–Whitney *U* = 355–451, *p* = 0.001–0.027, two-tailed). These results indicate that SBAC amended to bioretention and rain garden soil may enhance the removal of dissolved HOCs and/or prevent leaching of already sorbed pollutants. Amendment of SBAC appears to have a particular positive effect on the removal of moderately hydrophobic HOCs (log *K*_ow_ = 4.02–4.35). The reason for this observation could be that the moderately hydrophobic compounds do not exhibit as strong sorption to SOM as compounds of higher log *K*_ow_, e.g. pyrene, which was shown in the batch adsorption tests (see the “Batch sorption tests of SBAC and SOM” section). The highly hydrophobic compounds are removed to a large degree by the SOM alone, whereas the less hydrophobic compounds are more mobile in the soil bed, and SBAC is an important complement to further reduce their effluent concentrations.

Bioretention and rain gardens are usually designed to be drained within 24 to 72 h to prevent saturated conditions in the medium. The bed is drained to provide storage for back-to-back rain events, suitable habitat for vegetation and wet–dry cycling to promote different removal processes (Grebel et al. 2013; LeFevre et al. 2014). In this study, however, a continuous flow system was used, i.e. saturated conditions, to facilitate the experimental procedure and promote optimum contact between media and synthetic stormwater. The soil in the columns can be considered “ideally packed”, and water is assumed to be “ideally distributed” over the soil bed (due to upward water flow, use of porous rocks to evenly distribute incoming water and bentonite clay to prevent preferential flow along column walls). All these factors contribute to optimizing the contact between water and soil, i.e. a “best case scenario”. In addition, by using continuous flow through the medium, crack formation due to drying is avoided; consequently, channelling is avoided. In operating bioretention and rain gardens, channelling along plant roots and preferential flow paths is inevitable, leading to more or less limited contact between water and soil, and hence to limited sorption of pollutants onto soil particles and SBAC. On the other hand, one of the most common maintenance concerns for bioretention and rain gardens is clogging of media caused by fine sediment and soil compaction. Plant roots are essential in macropore formation in the media, helping to maintain the infiltration rate, aerate the soil and minimize compaction (Barrett et al. 2013).

#### The effect of dissolved organic matter on sorption capacity

**Fig. 2:**
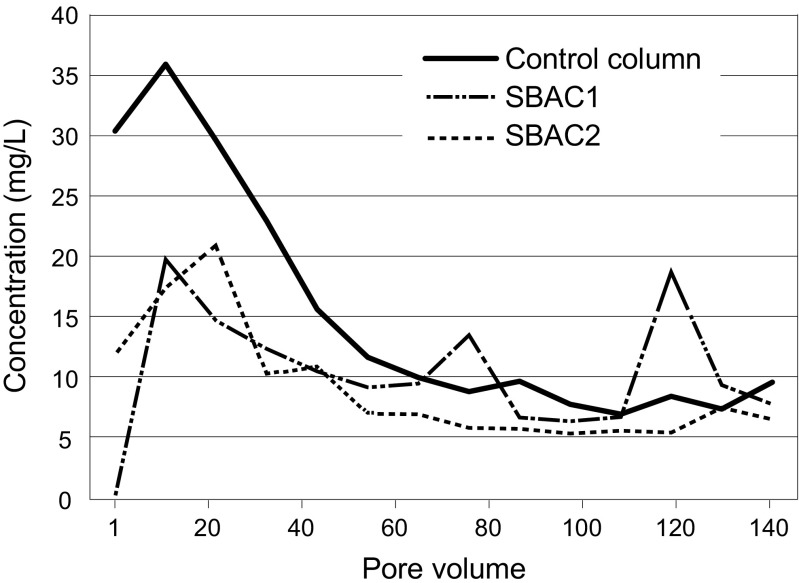
Concentrations of dissolved organic carbon (DOC [mg/L]) in effluent water from columns with added SBAC and control column without SBAC in the first 140 pore volumes (7 days) of the experiment. Each pore volume corresponds to 227 mL. No DOC was added to the columns; the graph illustrates DOC leached from the soil

In a previous study, the maximum adsorption capacity of SBAC was estimated to be at least 85 mg/g for a mixture of HOCs (Björklund and Li 2017). In the current study, the applied load of HOCs to each column during the course of the experiment corresponds to approximately 5.25 mg HOCs per g of SBAC, i.e. only 6% of the expected maximum adsorption capacity. Comparably low sorption of HOCs to SBAC in columns may be attributed to competition from organic matter leaching from the soil bed, observed primarily at the startup of the column experiments (Fig. 2). Soil organic matter contains a large portion of soluble, transformed material, called humic substances (Kyoichi 1987), which include both hydrophobic domains (e.g. non-polar aliphatic and aromatic moieties) and hydrophilic functional groups (e.g. polar phenolic and carboxylic moieties). The non-polar surface of activated carbon may interact with the non-polar part of humic substances. Efficient sorption of humic substances to activated carbon was shown in previous studies (Daifullah et al. 2004; Kilduff et al. 1996). We hypothesize that humic acids in the synthetic stormwater (20 mg DOC/L) and SOM released from the soil (≤80 mg DOC/L) lead to early saturation of the SBAC, and hence lower HOC adsorption than expected based on a previous study (Björklund and Li 2017). Unfortunately, HOC and DOC effluent concentrations could not be correlated since the DOC instrument was not able to give correct results for some of the batches run. This problem was discovered half-way through the experiment when many of the previous samples had already been discarded or were too old to be re-analysed.

Another column study was set up following the experiments with HOCs, to study the release of DOC from the soil bed (with a new instrument used for DOC analysis). The set-up and the soil used were exactly the same as in the described column tests (see the “Column tests” section), with the exception that tap water (not synthetic stormwater) was fed to the columns. Figure 2 shows that the release of DOC was elevated at the beginning of the experiment, and stabilized at <5 mg/L from pore volume 140 until the end of the experiment (35 days). This observation was expected, as less and less soluble organic matter was available for leaching from the soil. This gradual decrease in DOC leaching over time was also observed in column tests of peat and compost (Kalmykova et al. 2010; Mullane et al. 2015), whereas operating bioretention and rain gardens receive new DOC inputs with stormwater and degraded plants. Figure 2 shows the difference in DOC effluent concentrations between the two columns with added SBAC (≤20 mg/L) and the control column without SBAC (≤35 mg/L). Effluent DOC minimum, average and maximum concentrations were higher for the control column compared to SBAC-amended columns. These results suggest that a large portion of the released SOM was adsorbed by SBAC, thereby occupying adsorption sites on the carbon surface and inhibiting the sorption of HOCs (Li et al. 2003; Newcombe et al. 2002). This hypothesis is strengthened by results from a previous study, showing that SBAC can adsorb at least 20 mg DOC (in the form of technical HA) per gram SBAC (Björklund and Li 2017).

Because the tested HOCs only have limited polarity (alkylphenols and phthalates), it may be assumed that these HOCs are more strongly bound to the non-polar SBAC than are humic substances. In fact, our previous studies show that when competition for adsorption sites on SBAC becomes a factor, hydrophobicity plays an important role in the adsorption process: More hydrophobic compounds are removed to a higher degree by SBAC than compounds with lower log *K*_ow_ (Björklund and Li 2017). Although SBAC may favour sorption of HOCs, abundant humic substances are strong competitors. Sorption of organic pollutants may, however, take place on both SBAC and SOM, as shown in the batch tests. Another possible pathway for organic pollutants in the columns are to bind to humic substances sorbed onto the SBAC surface, and thereby become immobilized (Wen et al. 2013). However, this hypothesis has not been tested in the current study.

### HOCs in polluted column soils

#### HOC concentrations and loads in column soil

**Fig. 3:**
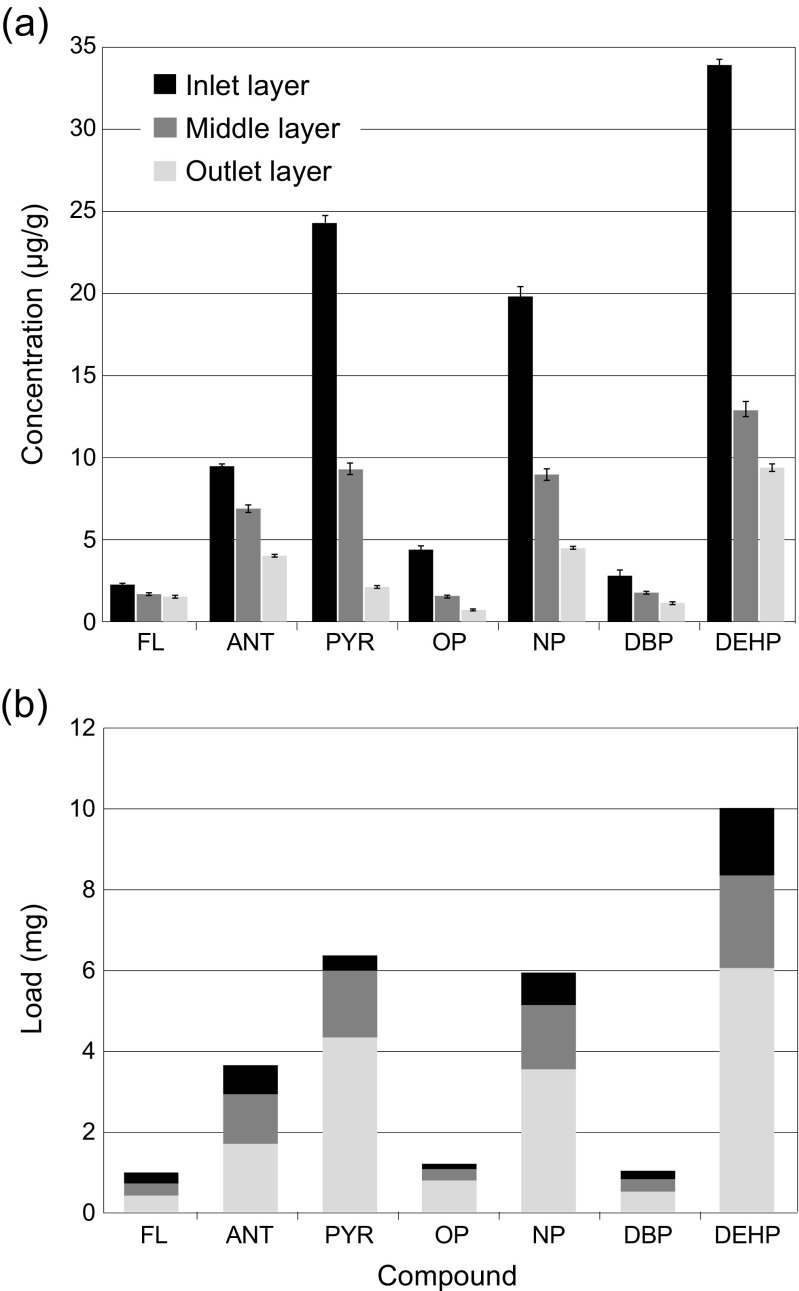
**a** Concentrations (μg/g) and **b** loads (mg) of HOCs in three separated soil layers (inlet layer 0–17 mm; middle layer 18–34 mm; and outlet layer 35–51 mm). *Errors bars* illustrate the standard error of the mean concentration in each layer (determined on triplicate samples)

As expected, the highest concentrations and the highest loads of HOCs were found in the soil layer closest to the column inlet (Fig. 3a and b, respectively). The HOC concentrations show strong correlation to compound log *K*_ow_ in the inlet and middle layer (Spearman’s *ρ* = 0.786 and 0.857, respectively), but only moderate correlation in the outlet layer (*ρ* = 0.536). Approximately 40–70% of the total load of each HOC was trapped in the first 17 mm of the soil, whereas 5–27% of the load was found in the layer closest to the outlet. The less hydrophobic compounds tend to migrate to a higher degree in the soil than the most hydrophobic compounds: Of all HOCs, fluorene exhibited the highest load portion in the layer closest to the outlet (27%), followed by DBP and anthracene (20%), whereas pyrene exhibited the lowest portion in the outlet layer (5%). This trend explains why HOC concentrations in the outlet layer were only moderately correlated to log *K*_ow_. These results compare to a field study on PAHs in rain gardens performed by DiBlasi et al. (2008): Most of the PAH retention appeared to take place in the surficial soil layers through sorption and particle capture. It should be noted that the rain garden had been in operation for less than 3 years when DiBlasi et al. (2008) performed their study. Similarly, Dierkes and Geiger (1999) found that PAH concentrations were generally highest in the upper 10 cm of roadside soil. However, at some sites, PAH concentrations did not decrease with soil depth (≤30 cm). Murakami et al. (2008) performed column infiltration studies with artificial stormwater and discovered that when the soil layer thickness was increased from 20 to 50 cm, the PAH removal efficiency increased from 17 to 56%, suggesting substantial vertical migration of PAHs. In addition, Strömvall et al. (2006) detected elevated concentrations of semi-volatile organic compounds and PAHs to a depth of 1.0 and 1.5 m, respectively, in soil sampled from road ditches. The authors proposed that this unexpected vertical transport of organic pollutants in the soil may be due to colloid-facilitated transport. In the current study, DOC in the form of technical humic acids was added to the synthetic stormwater, together with HOCs. The contact time between HAs and HOCs before application to soil columns ranged between 0 and 12 h (batches of stormwater were prepared twice a day). This may be less than the time needed for dissolved HOCs to equilibrate with HA colloids to form complexes (van de Kreeke et al. 2010). Assuming that the contact time is too short to form HA–HOC colloids to the same extent as in natural stormwater (Brown 2002; Kalmykova et al. 2013), the colloid-facilitated transport, i.e. the migration, of HOCs is underestimated in the column tests. The current study (Fig. 3), together with results from Strömvall et al. (2006) and Murakami et al. (2008), suggests that migration of HOCs through the medium bed (usually 0.5 m) may occur over the lifetime of a bioretention or rain garden. Furthermore, greater migration can be expected for less hydrophobic organic pollutants that are dissolved or for HOCs attached to colloids, since they may not be sorbed to or physically retained by the soil particles.

Both octylphenol and DBP exhibited lower loads in the polluted soil than expected from their respective log *K*_ow_ values (Fig. 3b). It is possible that the compound groups (PAHs, phenols and phthalates) sorbed with different intensities to the SOM depending on various physical–chemical and steric characteristics. However, there were too few compounds in each group to draw any conclusions on this matter. The total soil load of each compound is negatively correlated to average concentrations in the columns’ effluent water (Spearman’s *ρ* = −0.600 to −0.771), provided that DEHP effluent concentrations are neglected.

The mass of HOCs sorbed to the soil make up only 10–50% of the calculated theoretical maximum mass, *m*_*ads*_ (Eq. 2), that can be sorbed to the soil based on the SOM content (Table 2). This suggests that the soil was not saturated with HOCs in the column experiments, so that further sorption was possible. In addition, *C*/*C*_0_ = 1 was not reached for any of the HOCs (Fig. 1, Fig. S2), strengthening this hypothesis. The theoretical remaining sorption capacity (Table 2) did not follow a strong trend with hydrophobicity or actual sorbed mass in soil, implying that sorption is not simply explained by SOM content or compound hydrophobicity.

**Table 2:** Experimentally determined and theoretical sorption capacity of soil; desorption of HOCs from polluted soil (all three layers) determined after filtration and centrifugation

	Experimentally determined vs. theoretical sorption			Desorption			
	Total sorbed mass (mg)^a^	Theoretical maximum sorbed mass, *m*_*ads*_ (mg)	Remaining theoretical sorption capacity (%)	Desorbed mass (μg), determined on *filtered* samples	Percentage desorption (% of total sorbed mass)	Desorbed mass (μg), determined on *centrifuged* samples	Percentage desorption (% of total sorbed mass)
Fluorene	0.974	4.24	77	45.7	4.7	62.7	6.4
Anthracene	3.63	7.28	50	49.7	1.4	91.6	2.5
Pyrene	6.35	17.2	63	74.9	1.2	222	3.5
Octylphenol	1.19	10.3	88	50.2	4.2	89.3	7.5
Nonylphenol	5.92	19.0	69	112	1.9	246	4.2
DBP	1.02	2.10	52	20.8	2.0	35.0	3.4
DEHP	9.99	99.5	90	181	1.8	628	6.3

^a^Data from Fig. 3b

#### Desorption of HOCs from polluted soil

The desorbed loads of HOCs, following the ISO/TS 21268-2 standard method, were compared to the total loads of HOCs in polluted column soil to determine the percentage of desorption (Table 2). In general, the percentage desorption of HOCs was low (1.2–4.7%) compared to many other desorption studies of organic pollutants in soil, and the HOC sorption to soil cannot be considered reversible (Delle Site 2001; OECD 2000). The desorbed loads (μg) were correlated to the total load of HOCs in soil (Pearson’s *r* = 0.702), i.e. the initial mass of HOCs available for desorption, and to compound log *K*_ow_ (Spearman’s *ρ* = 0.631).

**Fig. 4:**
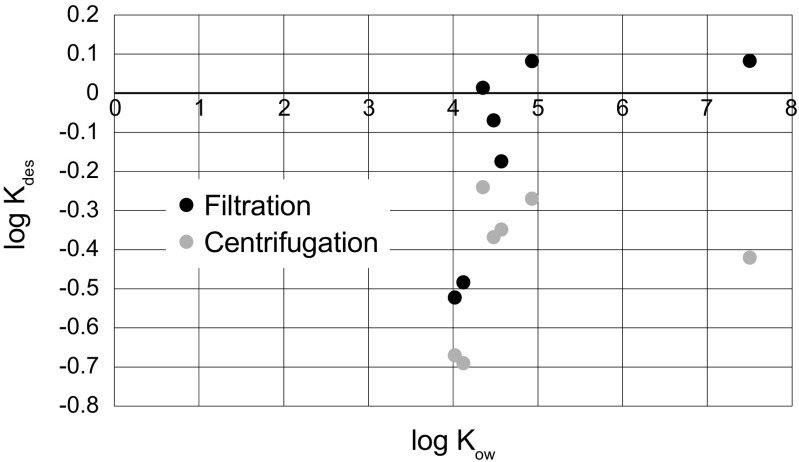
Logarithm of desorption coefficient, log *K*_*des*_, related to the hydrophobicity, log *K*_ow_, of the seven organic compounds investigated. The relationship is shown for filtered (1.5 μm pore size) and centrifuged (2000×*g*, 10 min) eluates

In the current study, the desorbed portion was centrifuged, followed by filtration (1.5 μm pore size), before extraction and analysis (according to ISO/TS 21268-2). For comparison, centrifuged samples (non-filtered) were also analysed for HOC concentrations. Centrifugation (2000×*g*, 10 min) led to separation of settleable solids. The TOC (total organic carbon) concentrations were 14.0 and 50.9 mg/L, respectively, for filtered and centrifuged samples. The difference in log *K*_*des*_ for HOCs between centrifuged and filtered samples is illustrated in Fig. 4. *K*_*des*_-values for DEHP were much lower (i.e. more mobile) than expected from the compound’s log *K*_ow_, especially in the centrifuged samples. In general, the desorbed HOC loads in centrifuged samples were 27% (fluorene) to 71% (DEHP) higher than in filtered samples (Table 2): This implies that a large portion of the most hydrophobic HOCs are sorbed to particles >1.5 μm (the filter pore size). Brown (2002) studied the partition of PAHs in stormwater particulate, colloidal and truly dissolved phases and came to the conclusion that, contrary to what can be expected from the log *K*_ow_ of PAHs, the importance of the colloidal phase decreased for the more hydrophobic compounds (log *K*_ow_ > 4.5) because the organic carbon in the particulate phase is a better sorbent. Based on this principle, we suggest that DEHP and other highly hydrophobic HOCs are principally sorbed to the organic matter in particles and that sorption to colloids is more important for HOCs of lower hydrophobicity. These results suggest that the cut-off for particle and/or colloid separation greatly affects outcome of desorption tests.

The least hydrophobic compounds fluorene and octylphenol exhibited the lowest *K*_*des*_ (Fig. 4), i.e. the most mobile compounds in soil. These data support the previously presented hypothesis (see the “Removal of HOCs in column tests” section), which the less hydrophobic HOCs exhibit weaker sorption to the SOM. Hence, SBAC plays a larger role in removal of these compounds in the column tests.

The main removal processes of organic pollutants in bioretention and rain gardens, adsorption and biodegradation take place mainly during wet and longer dry periods, respectively (LeFevre et al. 2014; Murakami et al. 2008). Zhang et al. (2014) showed that dry periods need to exceed at least 10 h for biodegradation to occur and that days to weeks may be necessary for degradation of compounds with low degradation rate. Consequently, in the continuous flow columns, sorption is promoted and little or no biodegradation is expected. Desorption data suggest that the HOCs are strongly bound to SOM (Table 2), resulting in low bioavailability and therefore limited opportunity for biodegradation (LeFevre et al. 2012b). Low desorption of HOCs also leads to limited regeneration of sorption sites and opportunities for other pollutants to find available sorption sites.

In planted bioretention and rain gardens, root exudates released from plants may play a role in the desorption of HOCs from soils. Root exudates contain substances such as organic acids, phenolic compounds, polysaccharides, and humic compounds, which are known to increase desorption of HOCs (Gao et al. 2010; LeFevre et al. 2013). Studies show that plant root exudates can abiotically alter and enhance desorption of the PAHs naphthalene, pyrene and phenanthrene from soil, although it is not known which mechanisms are behind the alteration. However, as highlighted by Grebel et al. (2013), the relevance of plant root exudates, and linked enhanced desorption and bioavailability, to stormwater contaminants and conditions is unknown.

### SBAC-enhanced sorption

As an example, fluorene was used to estimate the supplementary sorption capacity of SBAC that may be added to amended soil (see the “Estimation of enhanced pollutant removal using SBAC” section). By adding 0.5% (*w*/*w*) SBAC to 1 m^2^ bioretention (0.5 m^3^ soil), almost 1 g fluorene can be removed, in addition to the load removed by soil. In the simulated area (Surrey, BC, Canada), each square metre of bioretention is assumed to annually receive 110 and 1200 m^3^ runoff in traffic and residential areas, respectively. This runoff is assumed to carry 110 mg (traffic) and 60 mg (residential) fluorene to 1 m^2^ of bioretention. Given these loads, 0.5% (*w*/*w*) SBAC amendment to 1 m^2^ (0.5 m^3^) bioretention may sorb as much fluorene as the accumulated fluorene load transported with 17 years (residential) and 9 years (traffic) of runoff volumes.

The synthetic stormwater used in the column studies is a much less complex solution than natural stormwater, which contains a wide spectrum of organic pollutants; oil and grease; metals such as Zn, Pb, Cu, Ni and Cd; nutrients; organic and inorganic colloids; and dissolved compounds, as well as particles. Particles and thereto bound pollutants are expected to be trapped in the very top layer of bioretention and rain garden media (DiBlasi et al. 2008; LeFevre et al. 2014; Murakami et al. 2008). However, many of the dissolved pollutants compete for adsorption sites on the SBAC and contribute to saturation of the media. This suggests that the column study is a “best-case scenario” with low competition for adsorption sites, and shorter saturation time of the SBAC should be expected for natural stormwater. Although based on simplified calculations of the supplementary sorption capacity added by SBAC, the results suggest that SBAC may extend the lifetime of a bioretention or rain garden, possibly by approximately 10–20 years, before media saturation occurs.

## Conclusions

Batch adsorption studies show that SBAC has a significantly higher adsorption capacity of selected hydrophobic organic compounds than soil organic matter: Approximately five to eight times higher HOC mass is sorbed to SBAC than to SOM. This difference is assumed to depend on the abundance of hydrophobic sorption sites, in addition to high surface area and great microporosity of SBAC. The column tests indicate, however, that SBAC-amended soil is more efficient in removing moderately hydrophobic compounds, e.g. fluorene, octylphenol, and anthracene, than very hydrophobic compounds. Desorption tests revealed that the HOCs are generally strongly sorbed to the soil particles; the higher the hydrophobicity, the stronger is the sorption to SOM. In addition, more hydrophobic compounds exhibited stronger sorption to particles >1.5 μm, whereas less hydrophobic compounds appear to have attached to small particles and colloids. These results all point towards the conclusion that less hydrophobic HOCs exhibit weaker sorption to the SOM, i.e. higher risk of migration through soil beds and that SBAC played a larger role in the removal of these compounds in the column tests. The addition of as little as 0.5% (*w*/*w*) SBAC may extend bioretention media lifetime by approximately 10–20 years before saturation occurs with the less hydrophobic compounds. However, HOCs are competing with organic colloids for SBAC sorption sites. Column tests revealed that a large portion of the released SOM is adsorbed by SBAC, which may lead to early saturation of SBAC sorption sites. In addition, organic colloids present in stormwater may sorb HOCs and facilitate their transport through the soil bed.

Future studies should focus on the migration of HOCs through the medium bed, as it determines the lifetime of the bioretention and rain garden before leaching of HOCs becomes unacceptable. Although the highest HOC concentrations and loads were found in the first (upper) centimetres of the soil bed, migration of pollutants through the bed cannot be ruled out due to poor pollutant–soil contact in operating systems and colloid-facilitated transport of HOCs through the soil, which is assumed to be of importance in natural stormwater where organic colloids and compounds have enough time to equilibrate and form complexes.

## Electronic supplementary material

ESM 1 (DOCX 33 kb)

## Acronyms

ANT: Anthracene
DBP: Dibutyl phthalate
DEHP: Di(2-ethylhexyl) phthalate
DOC: Dissolved organic carbon
DOM: Dissolved organic matter
FL: Fluorene
HA: Humic acid
HOC: Hydrophobic organic compound
NP: 4-Nonylphenol
OP: 4-*tert*-Octylphenol
PAH: Polycyclic aromatic hydrocarbon
PYR: Pyrene
SBAC: Sewage sludge-based activated carbon
SOM: Soil organic matter
