# Supplementary material for: Removal of organic contaminants in bioretention medium amended with activated carbon from sewage sludge
Source: Environ Sci Pollut Res Int. 2017 Jun 29;24(23):19167–80. doi: 10.1007/s11356-017-9508-1 (PMC5556136; doi:10.1007/s11356-017-9508-1)
Supplement: Supplementary file 1 — (DOCX 33 kb) [file 11356_2017_9508_MOESM1_ESM.docx]

Supplementary Material

**Article title:** Removal of Organic Contaminants in Bioretention Medium Amended with Activated Carbon from Sewage Sludge

**Journal name:** Environmental Science and Pollution Research

**Author names:** Karin Björklund* and Loretta Li

*** Corresponding author’s affiliation:** Department of Civil and Environmental Engineering, Chalmers University of Technology, Sven Hultins Gata 8, 41296 Göteborg, Sweden; [karin.bjorklund@chalmers.se](mailto:karin.bjorklund@chalmers.se)

# **S1. Physical and Chemical Characteristics of Soil Medium**

Full details of physical and chemical characterisation of the soil used in this study, including eluate quality from soil leaching tests (e.g. metals, nutrients, dissolved organic carbon), are published in Björklund and Li (2016). Determination of pH (4.3), cation exchange capacity (6.60 meq/mL), particle size distribution (Fig. S1), particle density (2.54 g/cm^3^), moisture content (12.3 %) and loss on ignition (3.75 %) was performed according to standard methods in Sheldrick (1984).

**Fig. S1** *Particle size distribution of rain garden soil, determined on triplicate samples through sieve analysis*

## References:

Björklund K. and Li L. Y. (2016) *Sorption of DOM and hydrophobic organic compounds onto sewage-based activated carbon*, Water Science and Technology, 74(9), 852-860, DOI: 10.2166/wst.2016.240

Sheldrick B. H. (1984) *Analytical Methods Manual*, Land Resource Research Institute, Research Branch, Agriculture Canada, Ottawa, Canada.

# **S2. Breakthrough Curves of Anthracene, Octylphenol and DBP**

#

**Fig. S2** *Breakthrough curves of anthracene (log K_ow_ = 4.35), octylphenol (log K_ow_ = 4.12) and DBP (log K_ow_ = 4.57), showing the effluent-inlet concentration ratio (C/C_0_) as a function of the number of pore volumes passed through two columns with added SBAC and a control column (no SBAC). Each pore volume corresponds to 227 mL. Note the different scales on the y-axis*
